# Structure-Based Discovery of a Selective KDM5A Inhibitor that Exhibits Anti-Cancer Activity via Inducing Cell Cycle Arrest and Senescence in Breast Cancer Cell Lines

**DOI:** 10.3390/cancers11010092

**Published:** 2019-01-15

**Authors:** Guan-Jun Yang, Chung-Nga Ko, Hai-Jing Zhong, Chung-Hang Leung, Dik-Lung Ma

**Affiliations:** 1 State Key Laboratory of Quality Research in Chinese Medicine, Institute of Chinese Medical Sciences, University of Macau, Macao 999078, China; yb67509@connect.um.edu.mo (G.-J.Y.); zhonghaijing88@gmail.com (H.-J.Z.); 2 Department of Chemistry, Hong Kong Baptist University, Kowloon Tong, Hong Kong 999077, China; 12208205@life.hkbu.edu.hk

**Keywords:** KDM5A, Jumonji domain, histone demethylation, protein-protein interaction, virtual screening, cell cycle arrest, cell senescence, breast cancer

## Abstract

Breast cancer is the one of the most frequent causes of female cancer mortality. KDM5A, a histone demethylase, can increase the proliferation, metastasis, and drug resistance of cancers, including breast cancer, and is thus an important therapeutic target. In the present work, we performed hierarchical virtual screening towards the KDM5A catalytic pocket from a chemical library containing 90,000 compounds. Using multiple biochemical methods, the cyclopenta[c]chromen derivative **1** was identified as the top candidate for KDM5A demethylase inhibitory activity. Compared with the well-known KDM5 inhibitor CPI-455 (**18**), **1** exhibited higher potency against KDM5A and much higher selectivity for KDM5A over both KDM4A and other KDM5 family members (KDM5B and KDM5C). Additionally, compound **1** repressed the proliferation of various KDM5A-overexpressing breast cancer cell lines. Mechanistically, **1** promoted accumulation of p16 and p27 by blocking KDM5A-mediated H3K4me3 demethylation, leading to cell cycle arrest and senescence. To date, compound **1** is the first cyclopenta[c]chromen-based KDM5A inhibitor reported, and may serve as a novel motif for developing more selective and efficacious pharmacological molecules targeting KDM5A. In addition, our research provides a possible anti-cancer mechanism of KDM5A inhibitors and highlights the feasibility and significance of KDM5A as a therapeutic target for KDM5A-overexpressing breast cancer.

## 1. Introduction

KDM5A, also referred to as JARID1A or RBP2, is a transcriptional repressor belonging to the class of Jumonji-C domain-containing lysine-specific demethylases (JmjC-KDMs) [1,2]. KDMs consist of 20 demethylases which can be classified into 6 subfamilies: KDM2-7 [3,4]. Among them, KDM5 subfamily including four highly related histone enzymes KDM5A, KDM5B, KDM5C and KDM5D can remove di- and tri-methylation marks from activated H3K4 and be involved in many biological process such as tumorigenesis and differentiation of hematopoietic stem cells (HSCs) [4,5]. KDM5A is linked with multiple human cancer types, including tumors of the breast, lung, prostate, and stomach [6,7,8,9,10]. Overexpression of KDM5A promotes the migration, angiogenesis, and drug resistance of tumors [8,10,11,12]. As a cancer-promoting gene, KDM5A also are reported demethylase-dependently inhibits tumor suppressor genes such as *p27* and *p16*, and thus impairs proliferation of cancer cells via inducing cell arrest and senescence [13]. Previous studies have shown that KDM5A increased breast cancer cell proliferation, while knockdown of KDM5A altered H3K4 methylation and induced apoptotic cell death [8,14]. Unfortunately, due to the high similarity of among KDM5A, KDM4s and other members of KDM5 family, only few KDM5A inhibitors have been identified to date with most of them lacking selectivity for KDM5A [12,15,16,17,18,19,20,21,22]. Therefore, the development of potent and selective KDM5A inhibitors for treating breast cancer is urgently needed.

Natural products offer a diverse array of chemical scaffolds with distinct activity profiles and relatively mild toxicity [23,24,25,26,27,28,29,30,31]. Meanwhile, virtual screening has received attention as a versatile and effective tool for early stage drug discovery and lead optimization [32,33,34,35,36,37,38]. In this report, we describe the discovery of a selective and potent small-molecule KDM5A inhibitor (compound **1**) using high-throughput virtual screening. Compound **1** could directly impair the demethylase activity of KDM5A through inhibiting the protein–protein interaction between KDM5A-tri-/di-methylated histone 3 and promoting the accretion of H3K4me3 and H3K4me2 *in vitro* and *in cellulo*. Moreover, compound **1** caused growth arrest, and induced cell senescence of breast cancer cells, rendering it as a potential lead compound for the development of more efficacious drug candidates against KDM5A-overexpressing breast cancer.

## 2. Results

### 2.1. Compound ***1*** Identified as a Novel KMD5A Inhibitor via In Silico Screening

**Figure 1:**
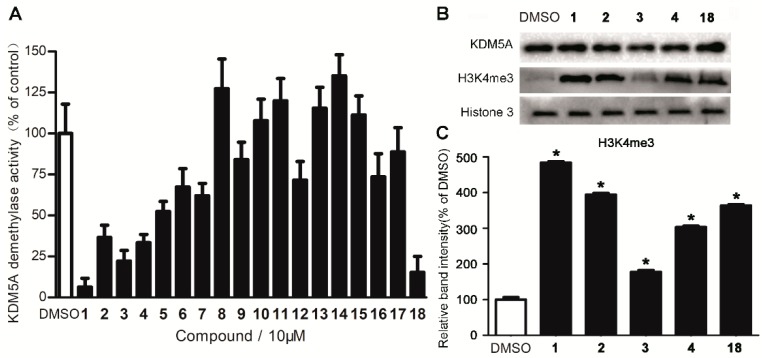
Identification of compound **1** as a novel KDM5A demethylase inhibitor. (**A**) KDM5A demethylase inhibition by compounds **1**–**18** in a chemiluminescence assay. (**B**) Compounds **1**–**4** and **18** inhibit KDM5A activity in MDA-MB-231 cells was revealed by Western blotting. (**C**) Densitometry analysis of H3K4me3 levels. Results are representative of three independent experiments. Data are represented as mean ± SD. Student’s *t* test, * p < 0.05.

To identify small molecules targeting KDM5A, 90,000 natural product or natural product-related structures from the ZINC compound library were screened *in silico* against the **18** (A well-known KDM5A inhibitor)-binding domain of KDM5A (PDB: 5CEH) using the internal coordinate mechanics (ICM) method [ICM-Pro 3.6-1d program (Molsoft, San Diego, CA, USA)] [12]. Based on the *in silico* results (Table 1), 17 compounds (**1**–**17**, Figure S1) were selected and purchased from commercial vendors, and then screened against KDM5A using an *in vitro* chemiluminescence assay (Figure 1A) [12]. Four hit compounds (**1**–**4**) showed greater than 50% inhibition activity and were further tested for KDM5A inhibition in MDA-MB-231 breast cancer cells, overexpress KDM5A (Figure 1B,C). From these assays, the ZINC33576 (compound **1**) emerged as a top candidate, showing higher potency than the positive control compound **18** both *in vitro* and *in cellulo*.

### 2.2. Compound ***1*** Is a Potent and Selective KDM5A Inhibitor

To further validate that KDM5A is the target of compound **1**, a KDM5A knockdown experiment was conducted in the MDA-MB-231 line. The results showed that incubation with either **1** or KDM5A siRNA significantly increased H3K4me3 and H3K4me2 levels (Figure S2A–D). To test if KDM5A is directly engaged by **1** *in cellulo*, the cellular thermal shift assay (CETSA) was conducted [39,40]. Compound **1** or **18** (3 µM) were incubated with MDA-MB-231 lysates for 30 min and the KDM5A content in the soluble fraction was detected by Western blotting (Figure S3A). KDM5A in lysates incubated with **1** or **18** showed significant thermal stabilization versus negative controls (Δ*T*_m_: 8.5 °C (**1**); 4.5 °C (**18**)) (Figure S3), indicating that compound **1** is able to target KDM5A even in the complicated cellular milieu. 

**Figure 2:**
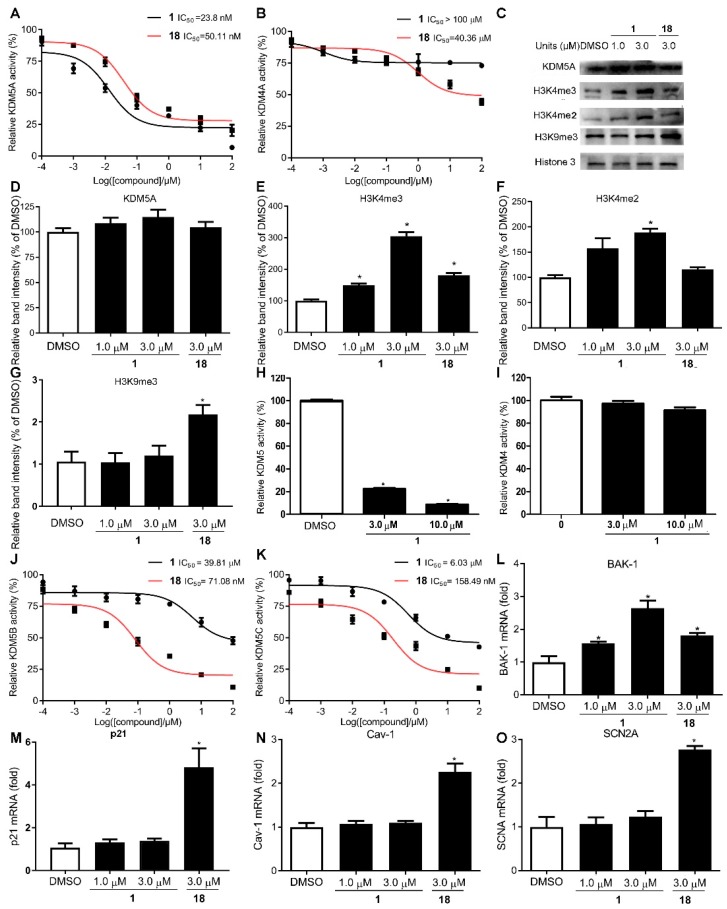
Compound **1** selectively inhibited KDM5A over both KDM4s and other members of KDM5 family. (**A**,**B**) **1** and **18** dose-dependently inhibits KDM5A (**A**) and KDM4A (**B**) demethylase activity *in vitro*; Effects of **1** and **18** on substrate levels of KDM5s and KDM4s *in cellulo* are detected by Western blotting (**C**–**G**), ELISA assay (**H**,**I**) and RT-qPCR (**L**,**M**); Effects of **1** and **18** on other members of KDM5 family *in vitro* and *in cellulo* are analyzed by chemiluminescence assay (**J**,**K**) and RT-qPCR (**N**,**O**). Data are represented as mean ± SD. Student’s *t* test, * *p* < 0.05.

Trimethylhomolysine (hKme3) is an analogue of *Nε*-trimethyllysine (Kme3). Histone demethylases KDM4A and KDM5A both recognized H3hK4me3 and H3K4me3 *in vitro* by isothermal titration calorimetry (ITC) [41], and most previously reported KDM5A inhibitors lack selectivity for KDM5A and KDM4s [42,43,44]. To verify the inhibitory potency and selectivity of **1** towards KDM5A over the highly similar KDM4s, an *in vitro* chemiluminescent assay was performed. Compound **1** selectively inhibited KDM5A demethylase activity over that of KDM4A (IC_50_ value: KDM5A vs. KDM4A: 23.8 nM vs. 100 μΜ) (Figure 2A,B). *In cellulo* assays were also carried out to further investigate the selectivity of compound **1** by comparing their specific translational and transcriptional substrate levels using western blotting, ELISA and RT-qPCR, the data revealed that exhibited dose-dependent inhibition against KDM5A demethylase activity versus KDM4s (Figure 2C–I,L,M). To explore whether compound **1** shows selectivity within the KDM5 family, CPI-455 (**18**), a selective inhibitor of KDM5 demethylases, lacking selectivity for the members of KDM5 was used as positive control [12], the chemiluminescent and RT-qPCR assays were administered, the results suggested that **1** exhibited much better selectivity for members of KDM5 family over **18** indicating by **1** comparing their IC_50_ values *in vitro (*Figure 2A,J,K) and the genes specifically transcriptional regulated by KDM5A (Bak-1) [45], KDM4A (p21) [46], KDM5B (CAV1) [47], and KDM5C (SCN2A) [48] *in cellulo* (Figure 2L–N). In summary, our data indicate that **1** is a strong and specific antagonist of KDM5A. 

### 2.3. In Silico Identification of Potentially Druggable Surface Sites on the KDM5A-H3K4me3-Binding Domain and Binding Mode of Compound ***1***

**Figure 3:**
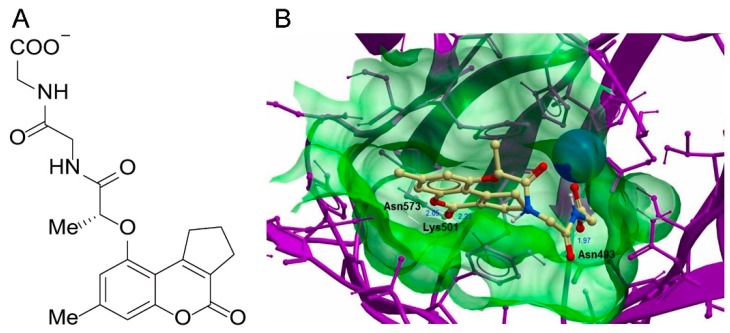
Minimized pose of **1** by *in silico* docking. (**A**) Molecule structure of compound **1**. (**B**) Protein KDM5A was displayed in the ribbon form. Compound **1** is depicted as a ball-and-stick model showing carbon (yellow), hydrogen (grey), oxygen (red) and nitrogen (blue) atoms. H-bonds are indicated as blue lines and the metal center as a green sphere. The binding pocket of the KDM5A is represented as a translucent green surface.

Molecular modelling was performed to understand the interaction of **1** with KDM5A. The most optimum conformation of compound **1** in complex with the structure of KDM5A_12–797_ showed that **1** could form a single interaction with the metal ion in the active site (Figure 3). Compound **1** was situated in the binding pocket of KDM5A, with its dipeptide chain extending deeply into a narrow groove of the protein. Moreover, one of the carbonyl oxygen atoms of the chain acts as a H-bonding acceptor with N493’s side chain, which is an interaction that has been described as being key in maintaining the conformation of KDM5A [16]. Additionally, the oxygen groups of the cyclopenta[c]chromen derivative of **1** form further hydrogen bond interactions with the side chains of both K501 and N573. A significant extent of shape complementarity between compound **1** and KDM5A is observed, indicating that this binding mode may also rely on substantial non-polar contacts, as for the reported KDM5 inhibitors **N10** and **N19 [12]**. Like compound **18**, the dipeptide chain moiety of compound **1** also locates snuggly in a crevice lined by Y409 and S478’s side chains, and the neighboring peptide chains of G410 and V473, giving little space left for additional functionality. The region the molecule occupies totally superposes with the interacting pose of 2-oxoglutarate (2-OG), suggesting a competitive mode of action.

### 2.4. Compound ***1*** Exhibits Potent Cytotoxicity Activity Against KDM5A-Overexpressing Breast Cancer Cells

**Figure 4:**
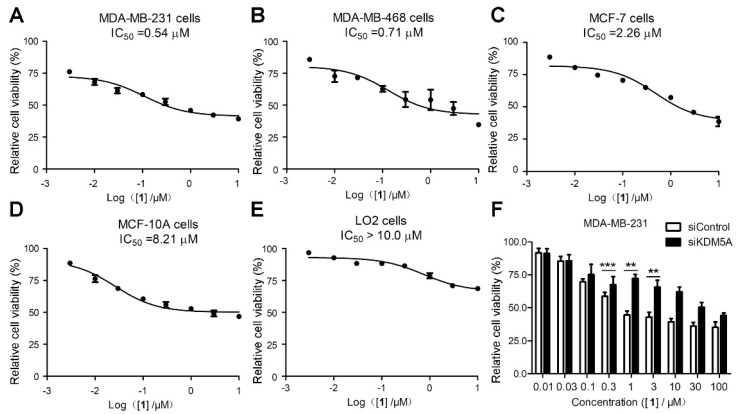
The cytotoxicity effect of compound **1** on breast cancer cells and normal cells. (**A**) MDA-MB-231 cells, (**B**) MDA-MB-468 cells, (**C**) MCF-7 cells, (**D**) MCF-10A, and (**E**) LO2 cells. Cells were exposed to the indicated concentrations of **1** for 72 h and cell viability was determined by MTT assay. (**F**) The cytotoxicity effect of **1** on siCon- or siKDM5A-treatement MDA-MB-231 cells. **1** inhibited the growth of on siCon- or siKDM5A-treatement MDA-MB-231 cells. Data are represented as mean ± SD. * *p* < 0.05, ** *p* < 0.01, *** *p* < 0.001 (Student’s *t* test).

KDM5A is upregulated in a variety of breast cancer cell lines [49]. Considering the potent activity of **1** at inhibiting KDM5A demethylase activity *in vitro* and *in cellulo* (Figure 2), compound **1** was further studied for its cytotoxicity in four breast cancer cell lines and one normal cell line using the MTT assay. MDA-MB-231, MDA-MB-468, and MCF-7 cells are KDM5A-overexpressing breast cancer cell lines, while the breast cancer cell line MCF-10A and the normal liver cell line LO2 express relatively low levels of KDM5A [45,49]. In the cytotoxicity assay, **1** exhibited potent cytotoxicity activity against MDA-MB-231, MDA-MB-468, and MCF-7 breast cancer cell lines (Figure 4A–C), while it exhibited relative low cytotoxicity against MCF-10A and LO2 cells (Figure 4D,E). The cytotoxicity activity of **1** towards MDA-MB-231 cells was further examined using the colony formation assay (CFA), which showed that **1** could significantly inhibited colony formation in a dose-dependent manner (Figure S4A). To study whether cytotoxicity effects of compound **1** were due to its inhibition of KDM5A, KDM5A were knocked down and the phenocopy by **1**-treatment were explored by MTT assay and CFA. The results showed that knockdown KDM5A significantly reduced compound **1**-induced cytotoxicity towards MDA-MB-231 cells (Figure 4F and Figure S4B) and upregulated the levels of tumor suppressor proteins p27 and p16 (Figure S2), which suggest that KDM5A is imperative for **1**-medited cytotoxicity. CPI-455 (**18**), a selective inhibitor of KDM5 demethylases, have been used to compare the effect of compound **1** through MTT assay. The data revealed that compound **1** have much higher cytotoxicity than **18** in four breast cancer cell lines and KDM5A inhibitors selectively inhibit breast cancer cell lines (Figure 4A–E and Figure S5). These results indicate that compound **1** is highly potent cytotoxicity to KDM5A-overexpressing breast cancer cell lines. 

### 2.5. Effect of Compound ***1*** on KDM5A-Mediated Transcriptional Activity

**Figure 5:**
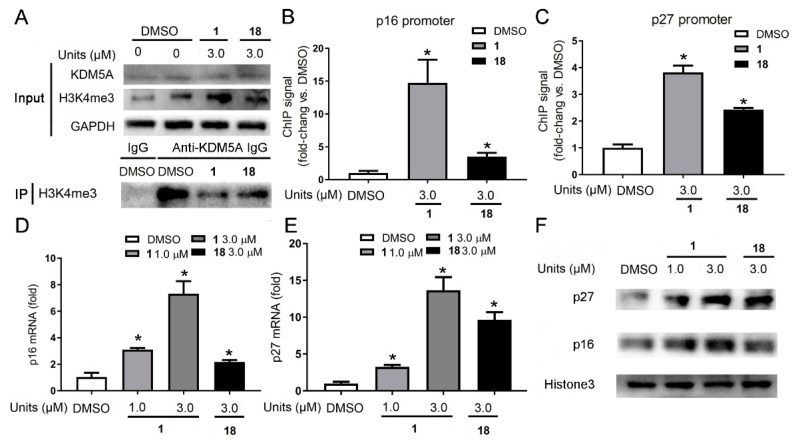
**1** and **18** promotes p16 and p27 production by blocking KDM5A-mediated H3K4me3 demethylation in MDA-MB-231 cells. Effect of **1** on the interactions KDM5A-H3K4me3 was examined by co-IP (**A**). Effect of **1** on H3K4me3 at promoters of *p16* and *p27* gene in MDA-MB-231 cells (**B**,**C**). ChIP assay was conducted with primary antibody against H3K4me3. transcriptional (**D**,**E**) and protein (**F**) levels of p21 and p27 in MDA-MB-231 cells treated with **1** or **18** were measured by Western blotting analysis. Bar graphs represented the mean enrichment relative to DMSO-treated groups and error bars reflect standard deviation of results derived from triplicate biological experiments. * *p* < 0.05, versus control (Student’s *t*-test).

Given the promising activity of **1** at antagonizing KMD5A demethylase, **1** was next studied for its mechanism of action *in cellulo*. The co-IP results revealed that **1** could increase H3K4me3 in a dose-dependent manner by disrupting the interaction between H3K4me3 and KDM5A (Figure 5A and Figure 2C). Previous reports have shown that KDM5A promoted cell proliferation in lung and gastric cancers by binding to the promoter of the genes *p27* and *p16*, resulting in the demethylation of H3K4me3 and direct repression of p27 and p16 [9,10]. Chromatin immunoprecipitation (ChIP) assay (primers in Table S1) was executed to further explore the effect of compound **1** on the binding of KDM5A to promoters in chromatin. After treatment with **1** for 24 h, MDA-MB-231 lysates were harvested and underwent cross-linking and immunoprecipitation with anti-H3K4me3 antibody. ChIP analysis revealed that **1** increased the amplification of the promoters of *p16* and *p27* gene (Figure 5B,C). Moreover, **1** could significantly elevate p16 and p27 at both transcriptional and translational levels (Figure 5D–F). These observations suggest that **1** could disrupt the recruitment of KDM5A to H3K4me3, increase trimethylation of H3K4 at the promoters of *p16* and *p27*, and thus raise their transcription in MDA-MB-231 cells, which is presumably mediated via its inhibition of KDM5A demethylase activity.

### 2.6. Anti-Proliferative Activity of Compound ***1*** Results from Inducing Cell Cycle Arrest and Cell Senescence

**Figure 6:**
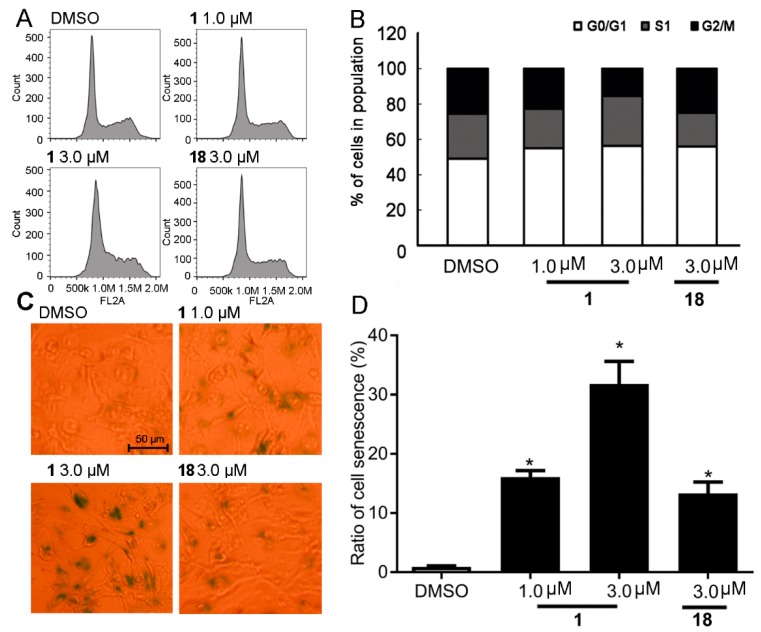
Compound **1** increases G1 population and induces cell senescence in MDA-MB-231 cells. Cells treated with **1** or **18** exhibit an increase entering G1 phase of cell cycle (**A**,**B**) and ratio of senescence cells (**C**,**D**) when compared with DMSO control. Data are represented as mean ± SD. Student’s *t* test, * p < 0.05. scale bar: 50 µm.

Knockdown of KDM5A altered H3K4 methylation and increased arrest at the G1 phase, via regulating expression of cell cycle genes including *p27* and *p16*, leading to senescence in cancer cells [9,10]. Based on the above results, cell-cycle analysis was conducted to further understand the mechanism of cytotoxicity of **1**. As expected, **1** could induce cell arrest of MDA-MB-231 cells at the G1 phase (Figure 6A,B) and cell senescence (Figure 6C,D) via upregulating cell cycle related proteins p16 and p27 (Figure 5F). Knockdown KDM5A also exhibited the similar phenocopy as **1** or **18** treatment indicating by increasing the levels of p16 and p27 proteins and ratio of senescence cells after KDM5A knockdown (Figures S2A,E,F and S6). KDM5A knockdown also impaired **1**-induced cell senescence indicating by no significant increase for ratio of senescence cells and levels of p16 and p27 compared DMSO group in KDM5A knockdown cells (Figure S6). Taken together, the cytotoxicity exhibited by **1** could be linked, at least partially, to the blocking of KDM5A activity *in cellulo* leading to G1 arrest and senescence induction via transcriptionally regulating p16 and p27 levels. 

## 3. Discussion

KDM proteins are overexpressed in a number of cancer types and have, in the last several years, become potential targets for anticancer therapy. Many KDM antagonists have been reported and several of these have been tested clinically [17,50]. KDM5A regulates the control of cell division and differentiation and is associated with inferior prognosis in different cancers [8,14]. There is intense interest in the identification of specific antagonists of KDM5A. For example, CPI-455 (**18**) is a commercially available compound that can inhibit the demethylase activity of KDM5A and suppress the proliferation of drug-sensitive tumor cells [12]. Meanwhile, derivatives of 3-thio-1,2,4-triazole (YUKA1 and YUKA2) were cytotoxic against several cancer cell lines with aberrant KDM5A expression [17]. However, KDM5 inhibitors identified thus few show only partial selectivity for KDM5. Thus, there is a pressing necessity to discover more selective KDM5A antagonists for the treatment of various types of cancers [12,16,17].

Natural/natural-like products provide a diverse array of biologically active scaffolds for developing novel drugs [23,24,25,26,27,28,29,30,31,51]. Meanwhile, *in silico* screening has become commonly used to accelerate the process of drug development [32,33,34,35,36,37,38,52,53,54]. This work reported the identification of the cyclopenta[c]chromen derivative **1** as an antagonist of KDM5A from a 90,000-compound chemical library using *in silico* screening. Similar compounds with cyclopenta[c]chromen skeletons have been reported as fungicides and antibacterial agents [51,52]. To the best of our knowledge, no pharmacological property of **1** has been presented before. 

*In silico* docking showed that the dipeptide chain and one of the carbonyl oxygen atoms of the chain of **1** play important roles in stabilizing the conformation of the KDM5A-**1** complex [12]. Significantly, *in vitro* and *in cellulo* experiments showed that compound **1** displayed significantly improved selectivity and potency against KDM5A activity compared to the positive control inhibitor **18**. Moreover, compound **1** exhibited much higher activity against KDM5A over the closely related KDM4s demethylase and other members of KDM5 family in *in vitro* and *in cellulo* assays, making it substantially more selective than existing KDM5A inhibitors reported in the literature. Therefore, **1** has the potential to overcome functional redundancies of KDM proteins in medical contexts. Moreover, our cytotoxicity assay results indicated that compound **1** could repress the growth of KDM5A-overexpressing breast cancer cell lines, with subsequent KD5MA knockdown experiments demonstrating that the anti-proliferative effect of **1** likely results from its inhibitory activity against KDM5A.

**Figure 7:**
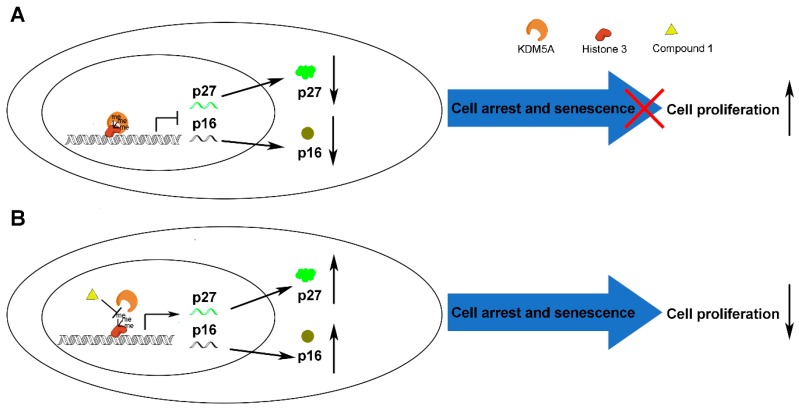
Proposed mechanism of how compound **1** induces anticancer effects in breast cancer cells. Compound prevents H3K4me3 and H3K4me2 from binding to KDM5A, resulting in the upregulation of H3K4me3-mediated gene transcription.

Mechanistically, **1** blocked the protein-protein interaction (PPI) between KDM5A and H3K4me3, resulting in the accretion of activated H3K4 and upregulation of p27 and p16 expression as demonstrated using ChIP, WB, and co-IP assays. This in turn induced cell cycle arrest in the G1 phase and cell senescence of KDM5A-overexpressing breast cancer cells (Figure 7). Taken together, these results highlight the potential of **1** to be used as a structural motif for developing KDM5A chemical probes and/or clinical inhibitors particularly against KDM5A-overexpressing breast cancer.

## 4. Materials and Methods

### 4.1. Cells and Reagents

Normal liver cells LO2 and breast cancer cell lines (MDA-MB-231, MCF-7, MCF-10A, and MDA-MB-468 cells) used here were cultured in Dulbecco’s modified Eagle’s medium (DMEM, Life Technologies, Carlsbad, CA, USA) supplemented with 10% fetal bovine serum and 1% penicillin and streptomycin. All cells were maintained at 37 °C with 5% CO_2_ in a humidified atmosphere. Compounds **1**–**17** (Commercially available, purity > 95%) were purchased from J&K Scientific Ltd. (Hong Kong, China). Compound **18** was bought from Medchemexpress (Shanghai, China). KDM5A Chemiluminescent Assay Kit was obtained from BSP Bioscience (San Diego, CA, USA). All the antibodies used in this article were shown resources in paper. All the compounds were dissolved in dimethyl sulfoxide (DMSO), MTT kit from Sigma-Aldrich (St. Louis, MO, USA).

### 4.2. Molecular Docking and Virtual Screening

The initial model of KDM5A in complex with **18** was derived from the X-ray crystal structure (PDB: 5CEH) using the molecular conversion procedure implemented in the ICM-pro 3.6-1d program (Molsoft). The molecular conversion procedure and high throughput molecular docking were administrated as previous reports [14,35].

### 4.3. KDM5A Inhibitor Screening Assay

The screening of KDM5A inhibitors was conducted by a multistep-reaction chemiluminescence-based method following the manufacturer’s instructions. Briefly, the tested compounds were dissolved in KDM5A assay buffer provided by the manufacturer and stored in −80 °C before use. To each test well, 20 µL of the diluted 1× KDM5A assay buffer [(50 mM HEPES-KOH (pH 8.0), 200 μM Fe(NH_4_)_2_(SO_4_)_2_, 1 mM α-ketoglutarate, 2 mM ascorbate)] [55], 5 µL 100 µM compounds **1**–**18**, and 25 µL of 1 ng/µL KDM5A were then added to wells pre-coated with histone substrate and incubated for one hour at room temperature. Subsequently, the wells were washed three times with TBST buffer and 100 µL of 400-fold diluted primary antibody was added into each well and incubated for 1 h. Finally, the wells were washed three times with TBST buffer, 100 µL diluted secondary HRP-labelled antibody was added, and the wells were sealed and incubated for 30 min at room temperature in the dark. Each well was washed three times with TBST buffer. Then, read plate within 30 min after adding 100 µL mixed luminescent substrates (1:1). The experimental results were qualified using SpectraMax M5 microplate reader (Molecular Devices, San Jose, CA, USA) under luminescence mode with typical 1 s integration time and 100 millisecond delays after plate movement.

### 4.4. KDM4A Activity and Inhibition Assay

The effect of compound **1** on KDM4A demethylase was detected as previously reported [56]. Briefly, 15 μL of assay buffer (20 mM Tris-HCl, pH 7.5, 20 mM sodium chloride), 10 μL of cofactor mixture (0.83 µg/µL), and 10 μL of 2.5 ng/µL KDM4A were added to each well, followed by the addition of 5 μL of DMSO or serially diluted compound **1** (0–100 μM). The reactions were initiated by adding 10 μL of the peptide solutions (200 μM) to the wells, except the background wells and the plate was incubated at 37 °C. After 30 min’s reaction, 40 μL of ammonium acetate and 10 μL of detector were added to the wells for cyclization to generate the fluorescent product, and the plate was incubated at 25 °C for another 15 min. Fluorescence intensity was monitored at excitation of 365 nm and emission of 465 nm in the SpectraMax M5 microplate reader (Molecular Devices).

### 4.5. Cellular Thermal Shift Assay

Cellular thermal shift assay was performed to monitor the target engagement of **1** in MDA-MB-231 cell lysates as in our previous report [56].

### 4.6. KDM5A Knockdown Assay

MDA-MB-231 cells were seeded in a 6-well plate at 80% confluences in medium for 24 h. Lipo3000 reagent, KDM5A siRNA (sense strand: 5′-GGCGGACGTTTCTTAAGAA-3′), and control siRNA (SC-35847, Santa Cruz Biotechnologies, Dallas, TX, USA) were gently mixed and incubated for 20 min at room temperature. Remove growth medium from cells and replace with 0.5 mL of fresh medium. Then the mixture 500 μL were added to each well. Cells were incubated at 37 °C in a CO_2_ incubator for 48 h post-transfection before the further research.

### 4.7. Cell Cytotoxicity and Proliferation Assay

The cell cytotoxicity and proliferation assay were detected by MTT assay and colony formation assay [57].

### 4.8. Western Blotting

The total cellular proteins were extracted with RIPA lysis buffer, and 20 mg were used. The membranes were probed with antibodies against p16^INK4^ and p27^kip1^ from Santa Cruz Biotechnologies, H3K4me3 (1:1000), H3K4me2 (1:1000), GAPDH (1:1000), and KDM5A (1:2500) from Abcam (Cambridge, MA, USA), followed by anti-mouse or rabbit horseradish peroxidase-conjugated immunoglobulin (Ig) G (1:1000) and developed with the enhanced chemiluminescent method. GAPDH signal served as a loading control. For immunoblotting of histone H3K4 mono-, di- and tri-methylation, we isolated a total histone fraction from nuclei using dilute acid extraction. Two micrograms of histone proteins were used and detected with antibodies against di- and tri-methylated H3K4 (Millipore, Billerica, MA, USA).

### 4.9. Cell Cycle Analysis

Propidium iodide staining was conducted for detection of DNA content. Following 3.0 μM **1** or **18** treatment for 6 h, harvested cells were washed in ice-cold PBS, fixed in 70% ethanol and stored at −20 °C overnight. Vehicle-treated cells were used as the control group. The MDA-MB-231 cell pellets were then incubated with RNase A (100 μg/mL) (Sigma, St. Louis, MO, USA), propidium iodide (50 μg/mL) (Sigma) and 0.05% Triton X-100. Cellular DNA content was detected on a FACSCalibur (BD Bioscience, Piscataway, NJ, USA) flow cytometer and analyzed by FlowJo_V10 software (Tree Star Inc., Ashland, OR, USA).

### 4.10. Cell Senescence Analysis

The cell senescence assay was conducted as previously report with minor modifications [58]. Briefly, the MDA-MB-231 cells were plated at 104 cells per well of a six-well plate in triplicate, and after 3 days SA-β-Gal activity was revealed with the senescence detection kit (KeyGEN BioTECH, Nanjing, China) and quantified (more than 200 cells per sample).

### 4.11. Co-Immunoprecipitation (co-IP) Assay

The co-IP assay was performed as previously described. Briefly, MDA-MB-231 cells were seeded at the density of 1 × 10^6^ cells in a six-well plate. Cells were treated with the indicated concentrations of **1** for 24 h under 37 °C 5% CO_2_. Cells were lysed and collected the protein samples. The concentration of protein samples was calculated using the Pierce BCA protein assay kit. 30 μg of each protein sample were incubated overnight with 10 μL pre-incubated anti-KDM5A magnetic beads according to the manufacturer’s protocol. The complex was washed 5 times to elute non-specific and non-cross-linked antibodies. Then, the precipitated proteins were subjected to SDS-PAGE and analyzed by Western blotting with anti-H3, anti-H3K4me2, and anti-H3K4me3 (1:1000, Abcam).

### 4.12. Chromatin Immunoprecipitation Assay

Chromatin immunoprecipitation (ChIP) assays were performed according to manufacturer’s protocols (Millipore) with slight modification. The anti-H3K4me3 IgG (1:50) (Abcam) was used to capture DNA fragments. Recovered DNAs were analyzed by real-time qPCR (ViiA™ 7 System, Life Technologies). The templates are prepared as previous procedure 6. Following was purified with ChIP DNA Purification Kit (Active Motif, Carlsbad, CA, USA). The recovered DNA was resuspended in TE buffer and used for the PCR amplification. The PCR primers for the target promoter are shown in Table S1.

### 4.13. Statistical Analysis

All statistical tests were conducted with GraphPad Prism version 5.0 (Graph Pad, San Diego, CA, USA). Statistical significance was determined using the Student’s *t*-test for experiments comparing two groups. Comparisons among groups were analyzed using analysis of variance (ANOVA). Unless stated otherwise, *p* values were 2-tailed and considered significant if *p* < 0.05. Error bars represent SEM of three experiments unless stated otherwise.

## 5. Conclusions

In conclusion, we have identified a potent and selective KDM5A inhibitors using structure-based virtual screening. Compound **1** displayed significantly improved selectivity and potency against KDM5A versus the reported inhibitor **18.** Mechanistically, **1** blocked the PPI of KDM5A and H3K4me3, and promoted p27 and p16 activation via raising H3K4me3 levels. Importantly, compound **1** showed much higher selectivity for KDM5A than KDM4A and other members of KDM5 family and is substantially more selective than existing KDM5A inhibitors reported in the literature. Additionally, compound **1** also reduced the growth of various breast cancer cell lines, possibly through inhibition of the KDM5A–H3K4me3 interaction and induction of G1 phase cell cycle arrest together with cell senescence. All the data presented here highlight the feasibility and significance of KDM5A as a therapeutic target for KDM5A-overexpressing breast cancer and demonstrate that **1** could function as a new motif for the generation of more selective and effcacious pharmacological candidates against KDM5A-overexpressing cancers.

## Figures and Tables

**Table 1:** Zinc number and docking scores of compounds used in this paper.

Name	ZINC No.	Relative Molecular Weight (Mr)	Scores
**1**	ZINC02140392	401.395	−30.01
**2**	ZINC02155003	442.448	−34.63
**3**	ZINC02113595	470.502	−32.43
**4**	ZINC08791298	441.463	−42.55
**5**	ZINC02091441	476.529	−31.4
**6**	ZINC01062498	364.445	−31.17
**7**	ZINC02113595	470.502	−32.43
**8**	ZINC12874897	523.633	−30.7
**9**	ZINC02128801	463.466	−33.14
**10**	ZINC03999938	378.472	−31.78
**11**	ZINC04012432	382.416	−30.26
**12**	ZINC12889905	418.406	−40.18
**13**	ZINC12893848	478.501	−33.87
**14**	ZINC08877996	468.509	−31.27
**15**	ZINC02334012	402.224	−30.58
**16**	ZINC08764396	439.423	−31.21
**17**	ZINC01475035	428.405	−34.84

## Supplementary Materials

The following are available online at http://www.mdpi.com/2072-6694/11/1/92/s1, Table S1: Primers for promoter specific ChIP-qPCR and RT-qPCR using in this paper. Figure S1: Chemical structures of compounds were evaluated in this study, Figure S2: KDM5A is the direct target of compound **1**, Figure S3: Compound **1** increases the thermal stabilization of KDM5A in cell lysates, Figure S4: Compound **1** inhibits cell growth as determined by a colony formation assay, Figure S5: The cytotoxicity effect of compound **18** on breast cancer cells and normal cells, Figure S6: KDM5A Knockdown reduces **1**- or **18**-induced cell senescence.
