# Supplementary material for: Structure-Based Discovery of a Selective KDM5A Inhibitor that Exhibits Anti-Cancer Activity via Inducing Cell Cycle Arrest and Senescence in Breast Cancer Cell Lines
Source: Cancers (Basel). 2019 Jan 15;11(1):92. doi: 10.3390/cancers11010092 (PMC6360022; doi:10.3390/cancers11010092)
Supplement: Supplementary file 1 [file cancers-11-00092-s001.pdf]

Supplementary Materials: Structure-Based Discovery of a Selective KDM5A Inhibitor that Exhibits Anti-Cancer Activity via Inducing Cell Cycle Arrest and Senescence in Breast Cancer Cell Lines

Guan-Jun Yang, Chung-Nga Ko, Hai-Jing Zhong, Chung-Hang Leung and Dik-Lung Ma

Supplementary Tables

Table S1. Primers for promoter specific ChIP-qPCR and RT-qPCR using in this paper.

Primers	Forward Primer	Reverse Primer	Applications
p27^{Kip1}	GCTCGTCGGGGTCTGTGTCTT	GGG CGAAGAGGTTCCTGCA	ChIP-qPCR
P16^{INK}	AGCACTCGCTCACGGCGTC	CTGTCCCTCAAATCCTCTGGA	ChIP-qPCR
GAPDH	AAAGGGCCCTGACAACTCTT	GGTGGTCCAGGGGTCTTACT	ChIP-qPCR
p27^{Kip1}	ATGTCAAACGTGCGAGTGTCTAA	TTACGTTTGACGTCTTCTGAGG	RT-qPCR
P16^{INK4}	TTCCTGGACACGCTGGT	CAATCGGGGATGTCTGAG	RT-qPCR
P21	GCGACTGTGATGCGCTAAT	TAGGGCTTCCTCTTGGAGAA	RT-qPCR
BAK-1	GCTCCCAACCCATTCACTAC	TCCCTACTCCTTTTCCCTGA	RT-qPCR
Cav-1	CCGCGACCCCAAGCA	CTGCAATCACATCTTCAAAGTC	RT-qPCR
SCN2A	GATGGGCAAGGTCAAGGTTG	ATTTGGCATCGATGAAGGGC	RT-qPCR
GAPDH	CCTGCACCACCAACTGCTTA	GGCCATCCACAGTCTTCTGAG	RT-qPCR

Supplementary Figures

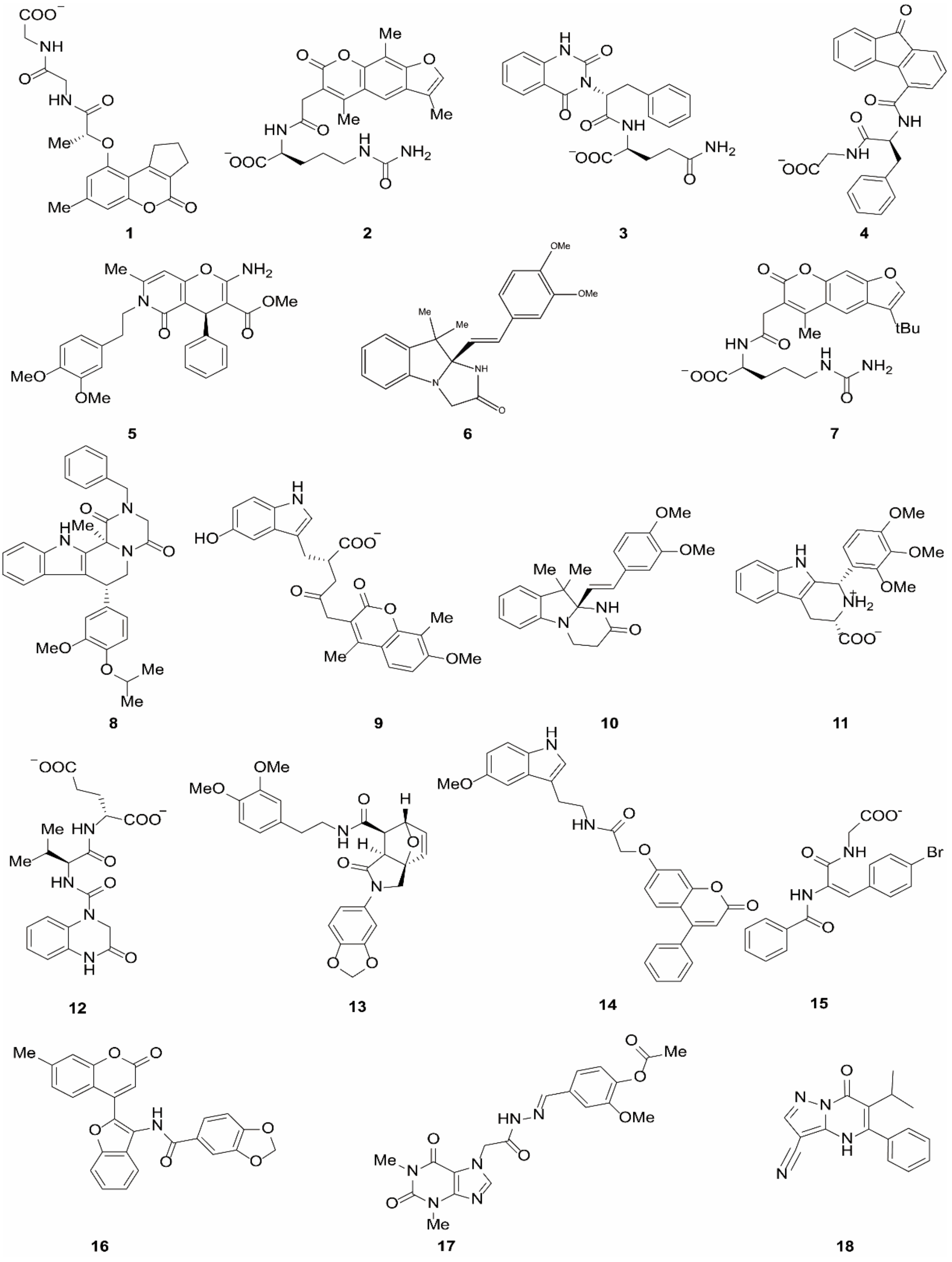

Figure S1. Chemical structures of compounds were evaluated in this study. **1**–**17**: Compounds were chosen to test by a preliminary chemiluminescence assay to evaluate their inhibition against KDM5A demethylase activity *in vitro*; **18**: positive control.

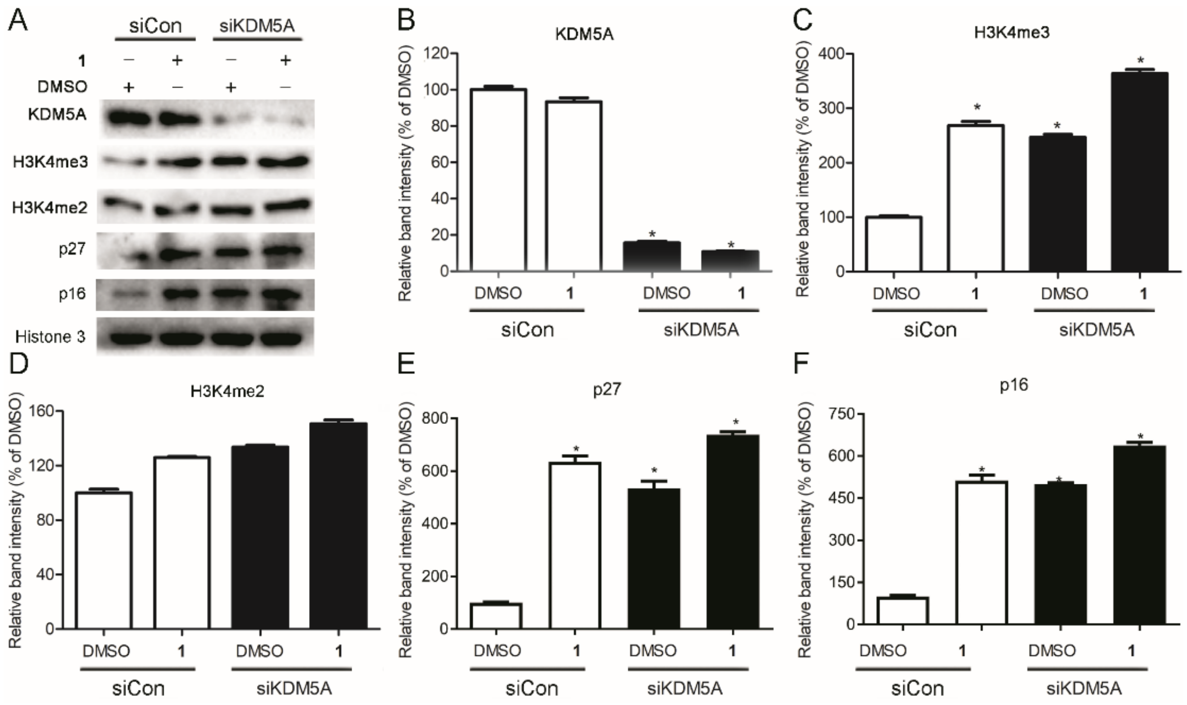

Figure S2. KDM5A is the direct target of compound **1**. (**A**) KDM5A siRNA treatment produces efficient target knockdown in MDA-MB-231 cells. KDM5A, H3K4me2, H3K4me3, p27, p16 and Histone 3 were blotted to control for total protein levels. (**B**–**D**) Relative densitometry analysis of KDM5A (**B**), H3K4me3 (**C**), and H3K4me2 (**D**), p27(**E**), and p16 (**F**). Results are representative of three independent experiments. KDM5A siRNA; Control siRNA: siCon. Data are represented as mean ± SD. Student's *t* test, * $p < 0.05$.

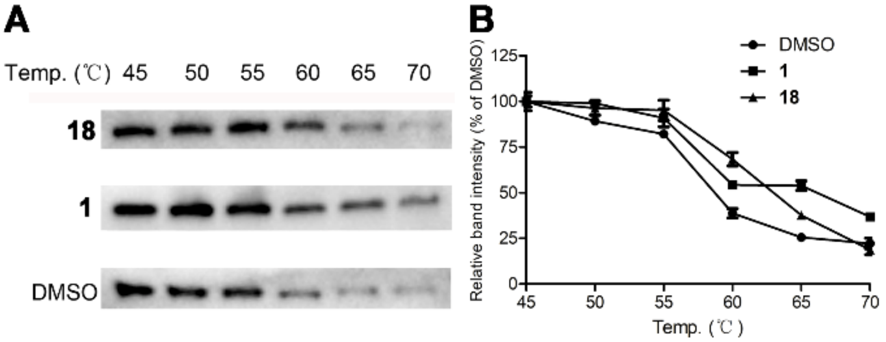

Figure S3. Compound **1** increases the thermal stabilization of KDM5A in cell lysates. (**A**) MDA-MB-231 cell lysates were treated with **1** and **18** at 3.0 μM. KDM5A content in the soluble fraction was detected by Western blotting. (**B**) Densitometry analysis of KDM5A content. Data are represented as mean ± SD. Student's *t* test, * $p < 0.05$.

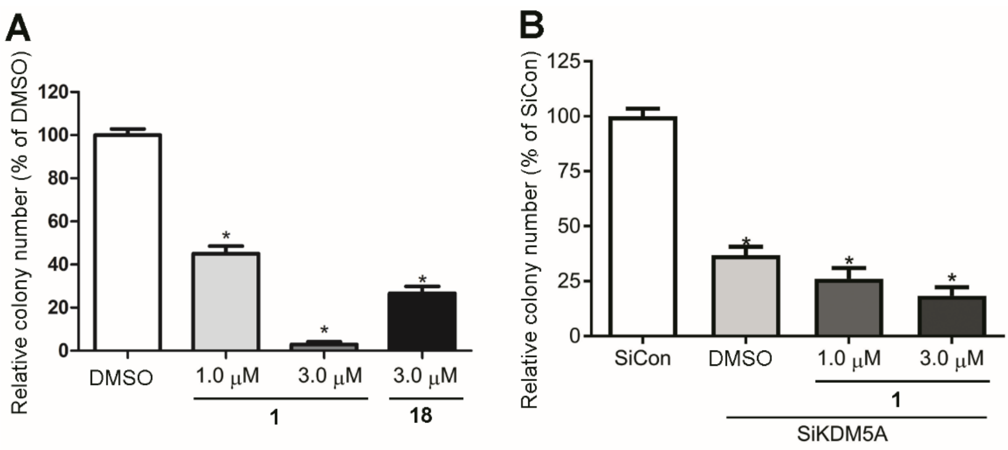

Figure S4. Compound **1** inhibits cell growth as determined by a colony formation assay. (**A**) Quantification of crystal violet-stained MDA-MB-231 cells (**B**) Quantification of crystal violet-stained SiCon- or siKDM5A-treatment MDA-MB-231 cells. Data are represented as mean ± SD. Student's *t* test, * $p < 0.05$.

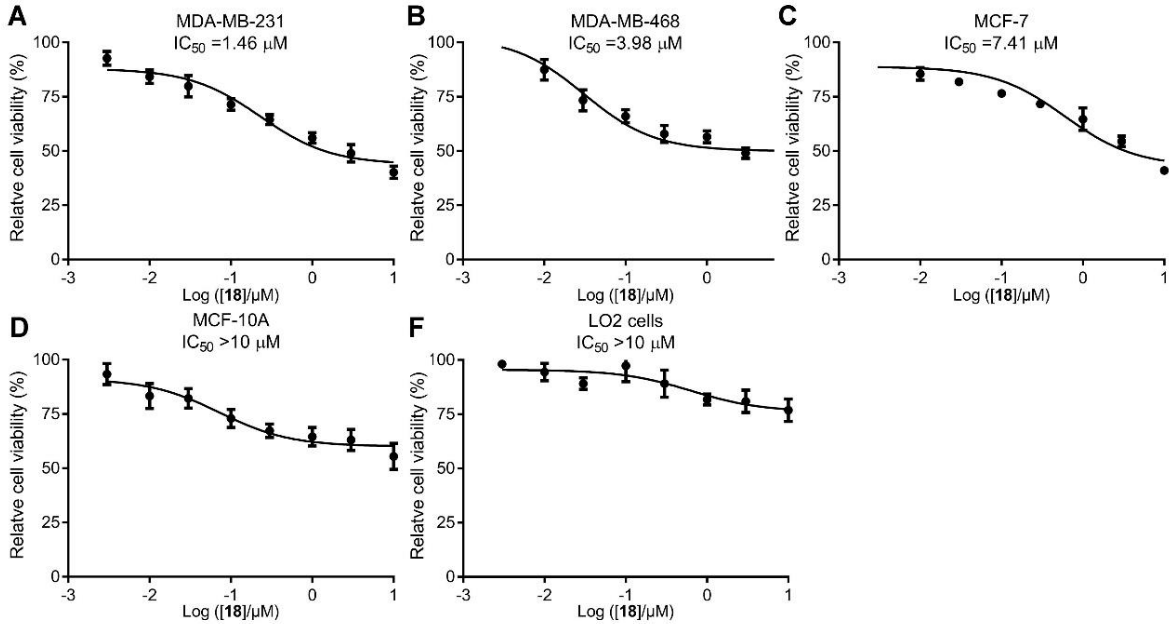

Figure S5. The cytotoxicity effect of compound **18** on breast cancer cells and normal cells. (**A**) MDA-MB-231 cells, (**B**) MDA-MB-468 cells, (**C**) MCF-7 cells, (**D**) MCF-10A, and (**E**) LO2 cells. Cells were exposed to the indicated concentrations of **18** for 72 h and cell viability was determined by MTT assay. Data are represented as mean ± SD.

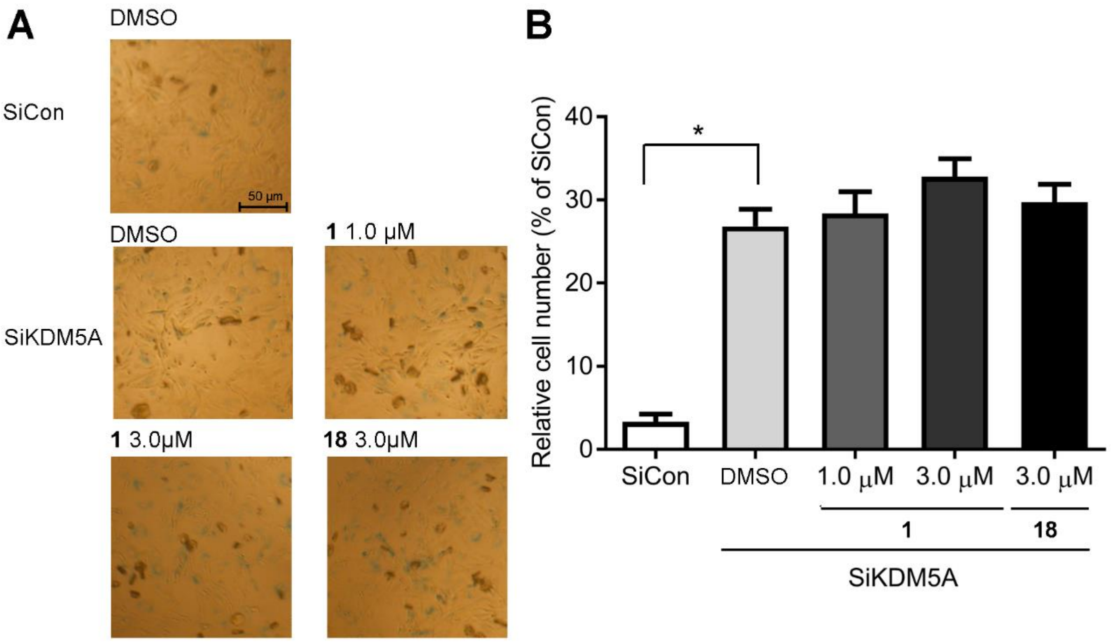

Figure S6. KDM5A Knockdown reduces **1**- or **18**- induced cell senescence. After 48 h treatment with siKDM5A or siCon, cells were treated with **1** or **18** 72 h and cell were stained with senescence detection kit. (**A**) Representative stained picture with different treatment. (**B**) Ratio of senescence cells (blue color) in (**A**) when compared with DMSO and siCon control. Data are represented as mean ± SD. Student's *t* test, * $p < 0.05$.

© 2019 by the authors. Licensee MDPI, Basel, Switzerland. This article is an open access article distributed under the terms and conditions of the Creative Commons Attribution (CC BY) license (http://creativecommons.org/licenses/by/4.0/).
